# Impacts of Temperature on the Stability of Tropical Plant Pigments as Sensitizers for Dye Sensitized Solar Cells

**DOI:** 10.1155/2014/739514

**Published:** 2014-02-23

**Authors:** Aiman Yusoff, N. T. R. N. Kumara, Andery Lim, Piyasiri Ekanayake, Kushan U. Tennakoon

**Affiliations:** ^1^Faculty of Science & Institute for Biodiversity & Environmental Research, Universiti Brunei Darussalam, Tungku Link, Gadong, BE1410, Brunei Darussalam; ^2^Applied Physics Program, Faculty of Science, Universiti Brunei Darussalam, Jalan Tungku Link, Gadong BE1410, Brunei Darussalam

## Abstract

Natural dyes have become a viable alternative to expensive organic sensitizers because of their low cost of production, abundance in supply, and eco-friendliness. We evaluated 35 native plants containing anthocyanin pigments as potential sensitizers for DSSCs. *Melastoma malabathricum* (fruit pulp), *Hibiscus rosa-sinensis* (flower), and *Codiaeum variegatum* (leaves) showed the highest absorption peaks. Hence, these were used to determine anthocyanin content and stability based on the impacts of storage temperature. *Melastoma malabathricum *fruit pulp exhibited the highest anthocyanin content (8.43 mg/L) followed by *H. rosa-sinensis* and *C. variegatum*. Significantly greater stability of extracted anthocyanin pigment was shown when all three were stored at 4°C. The highest half-life periods for anthocyanin in *M. malabathricum*, *H. rosa-sinensis,* and *C. variegatum* were 541, 571, and 353 days at 4°C. These were rapidly decreased to 111, 220, and 254 days when stored at 25°C. The photovoltaic efficiency of *M. malabathricum *was1.16%, while the values for *H. rosa-sinensis *and *C. variegatum *were 0.16% and 1.08%, respectively. Hence,* M. malabathricum* fruit pulp extracts can be further evaluated as an alternative natural sensitizer for DSSCs.

## 1. Introduction

Dye sensitized solar cell (DSSC) is a new derivative of a solar cell, developed by Grätzel [1]. It is based on semiconductor electrode-adsorbed dye, a counter electrode, and an electrolyte containing iodide and triiodide ions [2]. This device is capable of generating energy by converting the light absorbed into electrical energy.

Numerous metal complexes and organic dyes have been used and utilized as sensitizers [3]. Previously, it has been reported that the highest efficiency from a metal as sensitizer has been achieved from a compound containing Ruthenium, with a total of 11-12% efficiency [4]. Recent findings have found that perovskite sensitized solar cells have achieved a power conversion efficiency of approximately 15% [5]. Although such results provide better efficiency and high durability, the advantages are often offset by their high cost of production, complicated synthetic routes, environmental impact, and the tendency to undergo degradation in presence of water [6].

In contrast, the natural organic dyes are widely available and involve simple preparation, nontoxic, and complete biodegradation [7]. The use of nontoxic natural pigments as sensitizer would definitely enhance the environmental and economic benefits of this alternative form of solar energy conversion [8]. Due to these reasons, natural dyes are becoming attractive inexpensive candidates for renewable energy resources. The natural dye sensitizer may still produce very low efficiency, but with continuous advanced studies and research, improvisation of the efficiency of DSSCs has become a reality and hopeful.

Anthocyanins are the most abundant, naturally occurring flavonoid pigments which often give a bright red, blue, or violet color to plant petals, fruits, and stems [9]. Sometimes, they are present in a range of tissues including roots, tubers, and stems [4]. Since anthocyanin shows the red to blue color of the visible spectrum, it is considered as one of the best sensitizers for wide bandgap semiconductors [3].

The performance of the cell mainly depends on the dye used as sensitizer [10]. Optimizing the structure of a natural dye is necessary to improve DSSC efficiency [4]. Although anthocyanin pigments are abundant in plants, isolated anthocyanin pigments are highly instable and degradable [11]. Their stability is affected by several factors including pH, storage temperature, and sunlight exposure levels [12]. Hence, it is important to evaluate the optimum conditions required to maintain the anthocyanin stability over a long period of time.

Storage temperature plays a critical role for anthocyanin stability [13]. Investigating the effects of storage temperature on anthocyanin degradation will be highly beneficial because one of the vital steps in the procedure of manufacturing DSSCs involves storage of the extracted pigments.

In this study, a range of plants grown in Brunei Darussalam were tested for anthocyanin pigments. Special emphasis was paid to study the stability of promising pigments stored under different storage temperature regimes. Potential dye extracts were further tested as natural sensitizers in DSSCs.

## 2. Materials and Methods

### 2.1. Plant Materials

Brightly red/purple colored plant parts (flowers, fruits, tubers, and leaves) were harvested to determine the presence of anthocyanin (Table 1).

### 2.2. Anthocyanin Extraction

The anthocyanin extractions of the above plant parts were made following the procedure described by Rodriguez-Soana and Wrolstad [14]. 5 g of each freshly collected plant samples was used to extract the anthocyanin pigments. The pigments were initially extracted using 150 mL of 70% ethanol (w/v%) and stored overnight at 4°C. On the following day, the extraction was mixed thoroughly by using a magnetic stirrer for two hours under air-conditioned room temperature (25°C). The extraction was filtered using Whatman's ashless 110 mm filter paper to remove any solid residues. Subsequently, the extracts were centrifuged at 4500 rpm using a Denley BS400 (UK) centrifuge machine for five minutes to separate all residues. Lastly the supernatant of the ethanolic extracts was gently mixed with equal volumes of petroleum ether to separate polar and nonpolar pigments. The final ethanolic extract was assumed to carry only the polar anthocyanin pigments. This component was carefully poured to a 10 mL glass bottle, tightly stoppered and wrapped in aluminum foil to avoid exposure to light and treatments for different temperature regimes.

### 2.3. Plant Screening for Anthocyanin Pigments

Screening of separated anthocyanin pigments was done by measuring their absorbance spectra using UV-vis spectrophotometer (Shimadzu UV-1800, Japan). Before the commencement of absorbance measurements, each of the samples was treated with 45 *μ*L of concentrated HCl [15]. This acidification process converts anthocyanin derivatives to anthocyanidin that gives absorption spectra in the region of 490–550 nm [11, 15, 16]. Plant extracts that showed higher absorption spectra were selected for further investigations to evaluate the impacts of varying temperature regimes. All measurements were done in three replicates per sample.

### 2.4. Determination of Anthocyanin Content

To finalize the sample selection for DSSCs, those extracts that showed the highest UV-vis absorbance reading were chosen, and their anthocyanin contents were determined following the pH differential method described by Giusti and Wrolstad [11]. The results were expressed as micrograms per gram fresh weight.

Anthocyanin content was calculated according to the following equation:

(1) $$\begin{array}{c} \;\text{Anthocyanin}\;\text{pigment}\;\text{content}=\frac{A\times \text{MW}\times \text{DF}\times 10^{3}}{\varepsilon \times L}, \end{array}$$

where *A* = (*A*_520nm_–*A*_700nm_) pH 1.0 − (*A*_520 nm_–*A*_700 nm_) pH 4.5, MW  (Molecular  Weight) = 449.2 g/mol for cyanidin-3-glucoside, DF = Dilution factor, *ε* = 26900 L mol^−1^ cm^−1^, 10^3^ is the factor for converting g to mg, and *L* is the assumed path length in cm.

Aliquots of plant extracts were brought to pH 1 and 4.5 and allowed to equilibrate for one hour. The absorbance of each equilibrated solution was then measured at 520 nm (*λ*_max_) and 700 nm for haze correction. Spectroscopic absorbance readings were repeated against 70% ethanol as the reference. All measurements were done in three replicates per sample.

The MW used in this formula corresponds to the predominant anthocyanin in the sample. In some cases, predominant anthocyanin in a material may be known and could be different from cyanidin-3-glucoside. However, throughout the years, there has been a lack of uniformity in the values of absorptivity of purified anthocyanin, mainly due to difficulties of obtaining pure crystalline anthocyanin in adequate quantities [11, 17]. Since there is a huge variety of anthocyanin spread in nature, it has been suggested that if the major anthocyanin is unknown, it can be expressed as cyanidin-3-glucoside because that is the most abundant anthocyanin in nature [11, 12, 17–20].

### 2.5. Impacts of Storage Temperature on Anthocyanin Stability

The anthocyanin extracts of *M. malabathricum, H. rosa-sinensis, *and* C. variegatum* were stored in a tightly stoppered glass bottle fully covered with aluminum foil to avoid exposure to light. Extracts were stored at three different storage temperatures, namely, 4°C, −20°C, and 25°C, to evaluate the stability during storage. In order to determine the anthocyanin contents, the spectroscopic absorbance of the extracts were initially determined for three consecutive days followed by weekly measurements over a period of four months from September 2012 to January 2013.

### 2.6. Degradation Rate of Anthocyanin during Storage

The first-order reaction constant rate (*k*) and half-life (*t*_1/2_) were calculated using the following equation [21]:

(2) $$\begin{array}{c} \ln\left(\frac{C_{t}}{C_{o}}\right)=-k\times t,\\ t_{1/2}=\ln\left(0.5\right)\times k^{-1}, \end{array}$$

where *C*_*o*_ is the initial monomeric anthocyanin content and *C*_*t*_ is the monomeric anthocyanin content after *t* minute storage at a given temperature.

### 2.7. Photovoltaic Test of DSSC

The preparations of TiO_2_ anode are described elsewhere [22]. The anodes were dipped in the dye extract for overnight at room temperature (25°C) and dried out [15]. The cell was assembled using Dyesol's Test Cell Assembly Machine with the Surlyn (50 *μ*m, Dyesol). The electrolyte solution containing tetrabutylammonium iodide (TBAI; 0.5 M)/I_2_ (0.05 M), acetonitrile, and ethylene carbonate (6 : 4, v/v) [16] was introduced through a predrilled hole in platinum counter electrode. The cell was kept under irradiation of about 3-4 h for light soaking.

Finally *I-V* characteristic of the DSSC was measured under 1 sun level (DYESOL Solar Simulator LP-156B). The effective irradiated area of solar cell was 0.25 cm^2^. The performance of DSSC sensitized with anthocyanin pigments extracted from *M. malabathricum, H. rosa-sinensis,* and* C. variegatum* was evaluated by short circuit current (*J*_sc_), open circuit voltage (*V*_oc_), fill factor (ff), and energy conversion efficiency (*η*).

The absorbance spectra of the dye adsorbed on TiO_2_ electrodes were also measured. Before the commencement of absorbance measurements, each of the TiO_2_ electrodes were dipped in the dye extract overnight at room temperature (25°C) and air dried.

## 3. Results and Discussion

### 3.1. Plant Selection for DSSCs

**Figure 1:**
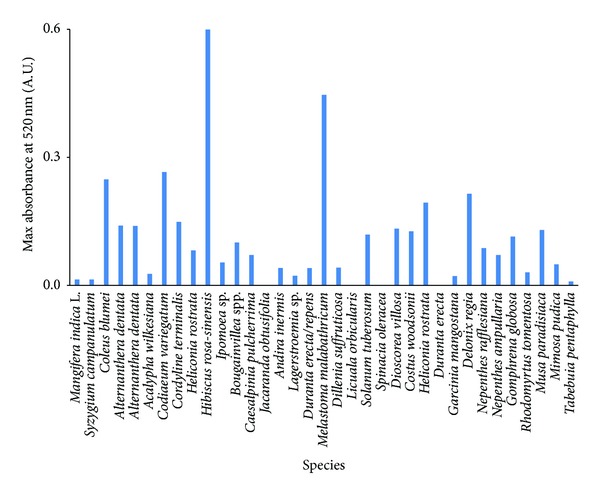
The absorbance spectra of anthocyanin pigments extracted from study species (*n* = 35) observed at 520 nm during the initial screening for the presence of anthocyanin pigments.

As shown in Figure 1, the maximum absorbance of anthocyanin varied significantly in different species. *Jacaranda obtusifolia, Licuala orbicularis, Spinacia oleracea*, and *Durantaerecta* flower extracts showed no absorbance at 520 nm; hence it can be concluded that they do not possess anthocyanin. Among the rest, 17 other plant extracts showed maximum absorbance of 0.1 or lower and therefore they were not selected to further investigations. On the other hand, the remaining sample extracts showed absorbance maxima greater than 0.1. However, only three species, each representing fruit, flower, and leaves (*Melastoma malabathricum, Hibiscus rosa-sinensis*, and *Codiaeum variegatum*), which showed that highest absorbance maxima were selected for further investigations.

### 3.2. Determination of Anthocyanin Content of Selected Plant Extracts for the Evaluation of DSSCs

Table 2 showed that among the samples investigated after preliminary screening, the highest anthocyanin concentration was found to be in the fruit pulp of *M. malabathricum* (8.43 mg L^−1^), followed by *H. rosa-sinensis* (4.63 mg L^−1^) then *C. variegatum* (2.22 mg L^−1^).

### 3.3. The Absorbance Spectrum

**Figure 2:**
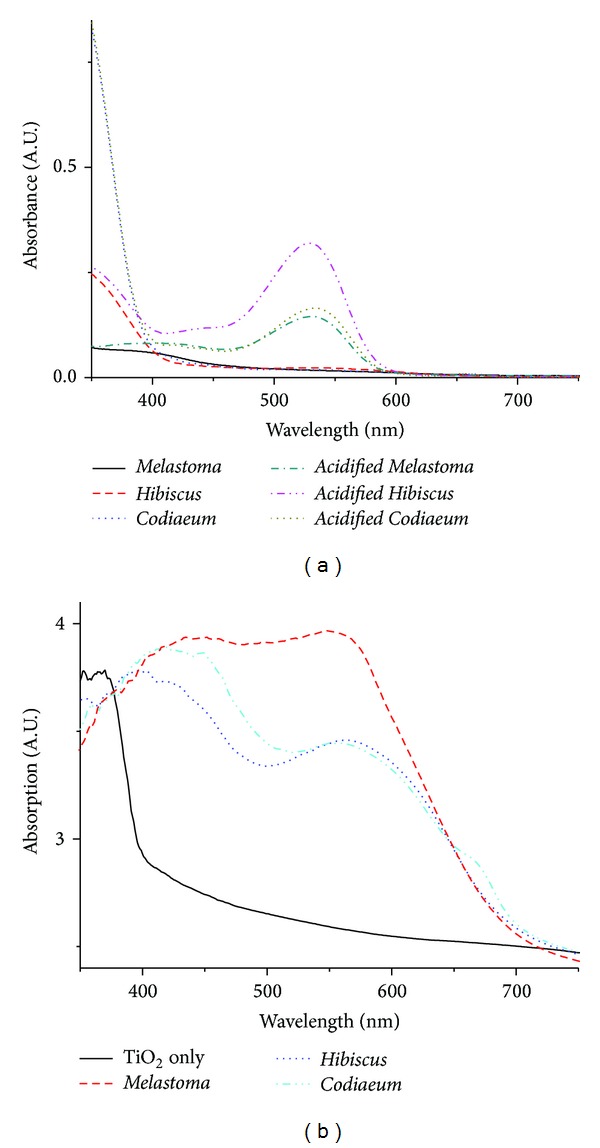
(a) The absorption spectra of the extracts of *Melastoma malabathricum, Hibiscus rosa-sinensis,* and *Codiaeum variegatum *in original and acidified extract and (b) absorption spectra of *Melastoma malabathricum*, *Hibiscus rosa-sinensis,* and *Codiaeum variegatum *dye onto TiO_2_ film.

All three extracts showed prominent peaks at 490–550 nm after the extracts were acidified with HCl (Figure 2(a)). This result indicated and proved once again that more anthocyanidin presents in the extracts [11, 15, 16].

On the other hand, Figure 2(b) showed that *M. malabathricum* extract exhibited the best absorbance after being adsorbed into the TiO_2_ electrode. This extract also gave the best efficiencies in DSSCs, while *C. variegatum in *TiO_2_ gave the second best absorbance, followed by *H. rosa-sinensis. *The absorbance results of the dye adsorbed TiO_2_ electrodes were consistent with *I-V* characteristics data.

### 3.4. The Effect of Storage Temperature on Anthocyanin Stability

**Figure 3:**
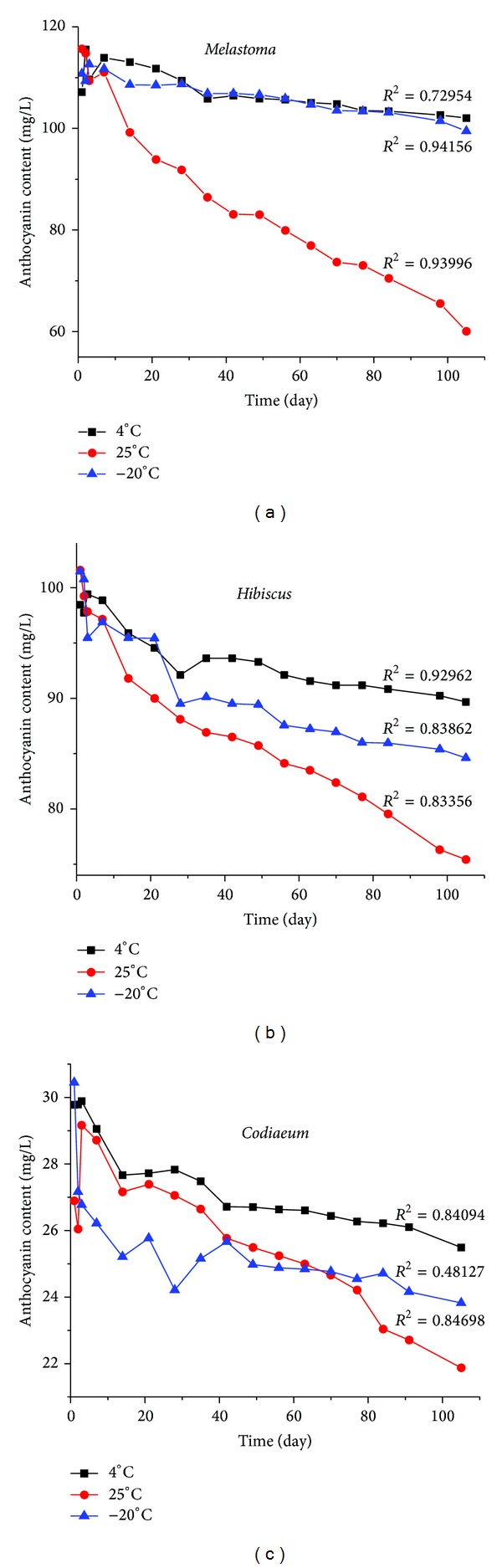
Degradation of anthocyanin pigments extracted from *M. malabathricum* fruit pulp (a),* H. rosa-sinensis* flowers, (b) and *C. variegatum* leaves (c) at three different storage temperatures (−20°C, 4°C and 25°C) over a three-month period.

The storage temperature had a strong influence on the degradation of anthocyanins extracted from all three extracts (see Figure 3 and Table 3).

The most distinctive pattern that was found in all three species was that anthocyanin pigments decreased progressively when stored at 25°C over a three-month period. However the stability of all three pigments was relatively high when the temperature was maintained at 4°C.

The degradation rates are represented by the half-life values; the higher the number, the more stable the anthocyanin extract. Result showed significantly greater stability of anthocyanin in all three species when they were stored at 4°C, and storage at 25°C resulted in much faster degradation. The highest half-life periods for anthocyanin in* M. malabathricum*, *H. rosa-sinensis,* and *C. variegatum* were 540.77, 571.19, and 352.86 days at 4°C, respectively, and it decreased rapidly to 110.71, 219.74, and 254.25 days at 25°C over a period of three months.

Similar results were reported by Janna et al. [23], who also studied the stability of *Melastoma malabathricum* and found that the suitable storage condition for anthocyanin pigment is acidic solution in dark and low temperature (4°C). The result of this investigation was also consistent with other similar studies where they found that anthocyanin pigments degrade faster as the temperature increases to 25°C and the stability is maintained at low temperatures (i.e., 4°C) [12, 21, 23].

A previous study on the anthocyanin degradation in black carrot showed that the *t*_1/2_ value in shalgam drinks maintained at 4 and 25°C were 34 and 11 weeks, respectively [24]. A similar study also found that the *t*_1/2_ value of monomeric anthocyanin of black carrot showed a distinct difference of 71.8 and 18.7 weeks, respectively, when maintained at 4 and 20°C, respectively [21]. Our investigation showed that frozen anthocyanin extracts maintained at −20°C also ensure a good stability over a period of three months; however, the best storage temperature was still 4°C.

### 3.5. The Efficiency of Natural Dye

**Figure 4:**
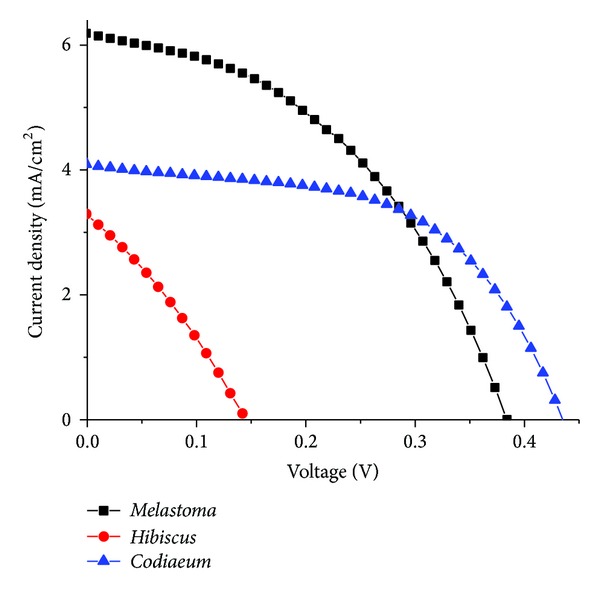
Current-voltage characteristics of the DSSCs sensitized with anthocyanins extracted from *Melastoma malabathricum*, *Hibiscus rosa-sinensis,* and *Codiaeum variegatum*.

The current-voltage characteristics of the DSSCs sensitized with the anthocyanin pigment extracted from *M. malabathricum* fruit pulp, *H. rosa-sinensis* flowers, and *C. variegatum* leaves are shown in Figure 4. The conversion efficiencies (**η**) of DSSCs were 1.16, 0.16, and 1.08%, respectively (Table 4). The highest effciency was obtained from DSSC sensitized with *M. malabathricum* fruit pulp extract with the open curcuit voltage (*V*_oc_ = 0.383 V), short curcuit current density (*I*_sc_ = 6.17 mA/cm^2^), and fill factor (ff = 0.44).

Natural pigments extracted from fruits and vegetables such as chlorophyll and anthocyanins have been extensively investigated as sensitizers for DSSCs. By far, the best performance reported was obtained from beet roots with an efficiency of 2.71% [25, 26].

Other studies include *Punicagranatum, Hibiscus sabdariffa*, pomegranate juice, wild Silicon prickly pear (*Opuntia vulgaris*), *Rhoeospathacea, *Mangosteen pericarp, red turnip, *Ficus reusa,* and *Hibiscus surattensis* with conversion efficiencies of 1.86, 1.6, 1.5, 2.06, 1.49, 1.17, 1.70, 1.18, and 1.14%, respectively [6, 7, 27–31].

Our study has shown that extract from *M. malabathricum* yielded the highest efficiency, 1.16%. The result is encouraging and the methods employed to maintain its stability is extremely promising. High efficiency obtained in the fruit pulps of* M. malabathricum* can be attributed to the carbonyl and hydroxyl groups of anthocyanin molecules present [3, 6, 7, 25]. This ability favours photoelectric conversion as it allows effective binding with the surface of TiO_2_ porous film. Further improvements in refinement of extraction and application methods will no doubt increase the efficiency of this dye in DSSCs.

## 4. Conclusion

Out of the 35 different species that were tested for the presence of anthocyanin pigments, *Melastoma malabathricum, Hibiscus rosa-sinensis*, and *Codiaeum variegatum* were selected as potential candidates in DSSCs. Among the three species, *M. malabathricum* extract exhibited the highest anthocyanin content. Based on the studies of anthocyanin stability on storage temperature, 4°C was the best to ensure pigment stability during storage. Among the three different species investigated, dye obtained from *M. malabathricum* fruit pulp also gave the highest efficiency. The photovoltaic performance of this dye was encouraging (1.16%). With further refinement of extraction and application methods, the efficiency of this dye can be further improved. Furthermore, due to the simple and cost-effective preparation techniques involved in the dye extraction of this species, it makes a promising alternative sensitizer for DSSCs.

## Figures and Tables

**Table 1:** List of plants studied to determine the anthocyanin content.

Number	Family	Species	Plant part analyzed for pigments
1	Anacardiaceae	*Mangiferaindica* L.	Leaves
2	Myrtaceae	*Syzygium * *campanulatum *	Leaves
3	Lamiaceae	*Coleus blumei *	Leaves
4	Amaranthaceae	*Alternantheradentata* var 1	Leaves
5	Amaranthaceae	*Alternantheradentata* var 2	Leaves
6	Euphorbiaceae	*Acalyphawilkesiana *	Leaves
7	Euphorbiaceae	*Codiaeumvariegatum *	Leaves
8	Agavaceae	*Cordylineterminalis *	Leaves
9	Heliconiaceae	*Heliconiarostrata *	Flowers
10	Malvaceae	*Hibiscus rosa-sinensis *	Flowers
11	Convolvulaceae	*Ipomoea* sp.	Flowers
12	Nyctaginaceae	*Bougainvillea* spp.	Flowers
13	Leguminosae	*Caesalpinia pulcherrima *	Flowers
14	Bignoniaceae	*Jacaranda obtusifolia *	Flowers
15	Papilionaceae	*Andirainermis *	Flowers
16	Lythraceae	*Lagerstroemia* sp.	Flowers
17	Verbenaceae	*Durantaerecta/repens *	Flowers
18	Melastomataceae	*Melastomamalabathricum *	Fruit pulp
19	Dilleniaceae	*Dilleniasuffruticosa*	Fruits
20	Palmaceae	*Licuala orbicularis *	Fruits
21	Solanaceae	*Solanumtuberosum *	Tubers
22	Amaranthaceae	* Spinacia oleracea*	Stem
23	Dioscoreaceae	*Dioscorea villosa *	Tubers
24	Costaceae	*Costuswoodsonii *	Flowers
25	Heliconiaceae	*Heliconiarostrata *	Flowers
26	Verbenaceae	*Durantaerecta *	Flowers
27	Clusiaceae	*Garciniamangostana *	Fruits
28	Fabaceae	*Delonixregia *	Flowers
29	Nepenthaceae	*Nepenthes rafflesiana *	Modified leaves
30	Nepenthaceae	*Nepenthes ampullaria *	Modified leaves
31	Amaranthaceae	*Gomphrenaglobosa *	Flowers
32	Myrtaceae	*Rhodomyrtustomentosa *	Flowers
33	Musaceae	*Musa paradisiacal *	Flowers
34	Leguminosae	*Mimosa pudica *	Flowers
35	Bignoniaceae	*Tabebuiapentaphylla *	Flowers

**Table 2:** Anthocyanin content of promising species that showed higher absorbance reading at 520 nm during the preliminary screening process.

Study species	Plant part used for pigment extraction	Anthocyanin content (mg/L fresh weight)*
*Hibiscus rosa-sinensis *	Flower	4.63
*Melastomamalabathricum *	Fruit pulp	8.43
*Codiaeumvariegatum *	Leaf	2.22

*n = 3.

**Table 3:** Kinetic parameters of anthocyanin degradation in *M*. *malabathricum* fruit pulp*, H*. *rosa-sinensis* flowers, and *C*. *variegatum* leaves at three different storage temperatures.

Species	Original pH	Temp./°C	*k*/10^−3^ (day^−1^)	*t*_1/2_ (day)
*Melastoma malabathricum *	pH 5.23	25	6.261	110.71
		4	1.282	540.77
		−20	1.286	539.13

*Hibiscus rosa-sinensis *	pH 5.73	25	3.154	219.74
		4	1.34	571.19
		−20	2.061	336.37

*Codiaeumvariegatum *	pH 5.93	25	2.726	254.25
		4	1.964	352.86
		−20	1.708	405.72

**Table 4:** The photoelectric parameters of DSSCs sensitized with natural dye extracted from the fruit pulp of *M*. *malabathricum*, *H*. *rosa-sinensis* flowers, and *C*. *variegatum. *

Sensitizer	*I*_sc_ (mA cm^−2^)	*V*_oc_ (V)	ff	*η* (%)
*Melastoma malabathricum *	6.17	0.383	0.44	1.16
*Hibiscus rosa-sinensis *	3.31	0.145	0.30	0.16
*Codiaeumvariegatum *	4.03	0.435	0.55	1.08

## References

[1] Grätzel M (2003). Dye-sensitized solar cells. *Journal of Photochemistry and Photobiology C*.

[2] Zhou H, Wu L, Gao Y, Ma T (2011). Dye-sensitized solar cells using 20 natural dyes as sensitizers. *Journal of Photochemistry and Photobiology A*.

[3] Hao S, Wu J, Huang Y, Lin J (2006). Natural dyes as photosensitizers for dye-sensitized solar cell. *Solar Energy*.

[4] Narayan MR (2012). Review: dye sensitized solar cells based on natural photosensitizers. *Renewable and Sustainable Energy Reviews*.

[5] Burschka J, Pellet N, Moon SJ (2013). Sequential deposition as a route to high-performance perovskite-sensitized solar cells. *Nature*.

[6] Hernandez-Martinez AR, Estevez M, Vargas S, Quintanilla F, Rodriguez R (2012). Natural pigment-based dye-sensitized solar cells. *Journal of Applied Research and Technology*.

[7] Zhou H, Wu L, Gao Y, Ma T (2011). Dye-sensitized solar cells using 20 natural dyes as sensitizers. *Journal of Photochemistry and Photobiology A*.

[8] Zhang D, Lanier SM, Downing JA, Avent JL, Lum J, McHale JL (2008). Betalain pigments for dye-sensitized solar cells. *Journal of Photochemistry and Photobiology A*.

[9] Młodzińska E (2009). Survey of plant pigments: molecular and environmental determinants of plant colors. *Acta Biologica Cracoviensia Series Botanica*.

[10] Wongcharee K, Meeyoo V, Chavadej S (2007). Dye-sensitized solar cell using natural dyes extracted from rosella and blue pea flowers. *Solar Energy Materials and Solar Cells*.

[11] Giusti MM, Wrolstad RE (2001). Characterization and measurement of anthocyanin by UV-visible spectroscopy. *Current Protocols in Food Analytical Chemistry*.

[12] Castañeda-Ovando A, Pacheco-Hernández MDL, Páez-Hernández ME, Rodríguez JA, Galán-Vidal CA (2009). Chemical studies of anthocyanins: a review. *Food Chemistry*.

[13] Patras A, Brunton NP, Tiwari BK, Butler F (2011). Stability and degradation kinetics of bioactive compounds and colour in strawberry jam during storage. *Food and Bioprocess Technology*.

[14] Rodriguez-Saona LE, Wrolstad RE (2001). Extraction, isolation and purifications of anthoyanins. *Current Protocols in Food Analytical Chemistry*.

[15] Kumara NTRN, Ekanayake P, Lim A, Iskandar M, Ming LC (2013). Study of the enhancement of cell performance of dye sensitized solar cells sensitized with *Nephelium lappaceum* (F: Sapindaceae). *Journal of Solar Energy Engineering*.

[16] Kumara NTRN, Ekanayake P, Lim A (2013). Layered co-sensitization for enhancement of conversion efficiency of natural dye sensitized solar cells. *Journal of Alloys and Compounds*.

[17] Lee J, Barnes KW, Eisele T (2005). Determination of total monomeric anthocyanin pigment content of fruit juices, beverages, natural colorants, and wines by the pH differential method: collaborative study. *Journal of AOAC International*.

[18] Dey PM, Harborne JB (1993). *Plant Phenolics Methods in Plant Biochemistry*.

[19] Francis FJ (1989). Food colorants: anthocyanins. *Critical Reviews in Food Science and Nutrition*.

[20] Kong J-M, Chia L-S, Goh N-K, Chia T-F, Brouillard R (2003). Analysis and biological activities of anthocyanins. *Phytochemistry*.

[21] Kirca A, Özkan M, Cemeroğlu B (2006). Effects of temperature, solid content and pH on the stability of black carrot anthocyanins. *Food Chemistry*.

[22] Ekanayake P, Kooh MRR, Kumara NTRN (2013). Combined experimental and DFT–TDDFT study of photo-active constituents of *Canarium odontophyllum* for DSSC application. *Chemical Physics Letters*.

[23] Janna OA, Khairul A, Maziah M, Mohd Y (2006). Flower pigment analysis of *Melastoma malabathricum*. *African Journal of Biotechnology*.

[24] Turker N, Aksay S, Ekiz HI (2004). Effect of storage temperature on the stability of anthocyanins of a fermented black carrot (*Daucus carota var. L.*) beverage: shalgam. *Journal of Agricultural and Food Chemistry*.

[25] Shahid M, Shahid-ul-Islam, Mohammad F (2013). Recent advancements in natural dye applications: a review. *Journal of Cleaner Production*.

[26] Sandquist C, McHale JL (2011). Improved efficiency of betanin-based dye-sensitized solar cells. *Journal of Photochemistry and Photobiology A*.

[27] Calogero G, Yum J-H, Sinopoli A, Di Marco G, Grätzel M, Nazeeruddin MK (2012). Anthocyanins and betalains as light-harvesting pigments for dye-sensitized solar cells. *Solar Energy*.

[28] Hug H, Bader M, Mair P, Glatzel T (2014). Biophotovoltaics: natural pigments in dye-sensitized solar cells. *Applied Energy*.

[29] Calogero G, Di Marco G, Cazzanti S (2010). Efficient dye-sensitized solar cells using red turnip and purple wild sicilian prickly pear fruits. *International Journal of Molecular Sciences*.

[30] Bazargan MH (2009). Performance of nano structured dye-sensitized solar cell utilizing natural sensitizer operated with platinum and carbon coated counter electrodes. *Digest Journal of Nanomaterials & Biostructures*.

[31] Lai WH, Su YH, Teoh LG, Hon MH (2008). Commercial and natural dyes as photosensitizers for a water-based dye-sensitized solar cell loaded with gold nanoparticles. *Journal of Photochemistry and Photobiology A*.

